# Joint Optimization of Multi-Hop Broadcast Protocol and MAC Protocol in Vehicular Ad Hoc Networks

**DOI:** 10.3390/s21186092

**Published:** 2021-09-11

**Authors:** Zhonghui Pei, Xiaojun Wang, Zhen Lei, Hongjiang Zheng, Luyao Du, Wei Chen

**Affiliations:** 1 School of Information Engineering, Wuhan University of Technology, Wuhan 430070, China; peizhonghui@whut.edu.cn; 2 School of Electronic Engineering, Dublin City University, 9PPR+4F Dublin 9, Ireland; xiaojun.wang@dcu.ie; 3 School of Automation, Wuhan University of Technology, Wuhan 430070, China; leizhen@whut.edu.cn (Z.L.); duluyao@whut.edu.cn (L.D.); 4 Shanghai Engineering Technology Research Center for Intelligent and Connected Vehicle Terminals, Shanghai 200030, China; hongjiangzheng@pateo.com.cn

**Keywords:** VANETs, MAC, contention window, Q-learning, two-hop neighbor, multi-hop broadcast

## Abstract

Beacon messages and emergency messages in vehicular ad hoc networks (VANETs) require a lower delay and higher reliability. The optimal MAC protocol can effectively reduce data collision in VANETs communication, thus minimizing delay and improving reliability. In this paper, we propose a Q-learning MAC protocol based on detecting the number of two-hop neighbors. The number of two-hop neighbors in highway scenarios is calculated with very little overhead using the beacon messages and neighbor locations to reduce the impact of hidden nodes. Vehicle nodes are regarded as agents, using Q-learning and beacon messages to train the near-optimal contention window value of the MAC layer under different vehicle densities to reduce the collision probability of beacon messages. Furthermore, based on the contention window value after training, a multi-hop broadcast protocol combined with contention window adjustment for emergency messages in highway scenarios is proposed to reduce forwarding delay and improve forwarding reliability. We use the trained contention window value and the state information of neighboring vehicles to assign an appropriate forwarding waiting time to the forwarding node. Simulation experiments are conducted to evaluate the proposed MAC protocol and multi-hop broadcast protocol and compare them with other related protocols. The results show that our proposed protocols outperform the other related protocols on several different evaluation metrics.

## 1. Introduction

Vehicular ad hoc networks (VANETs) need to satisfy reliable communication requirements for intelligent transportation systems (ITS) and unmanned vehicles. The messages transmitted in the network are mainly service messages and security messages, which are respectively transmitted on the service channel (SCH) and the control channel (CCH). Security messages have higher requirements for low transmission delay and high communication reliability, bringing many challenges to VANETs [1]. Beacon messages and emergency messages transmitted by broadcast in VANETs are all security messages. Through periodic single-hop broadcast beacon messages, vehicles provide neighboring vehicles with their status information, such as location, speed, acceleration and deceleration, overtaking, and so on. When a traffic accident occurs, a vehicle involved in the accident needs to notify other vehicles in a more extended range through emergency messages by multi-hop broadcast to avoid risks in time.

To support the message transmission service between vehicles, IEEE and 3GPP respectively proposed the wireless access in vehicular environments (WAVE) standard and the cellular vehicle-to-everything (C-V2X) standard, which use different channel access technologies. This paper is based on WAVE standard system, including IEEE 802.11p and the family of IEEE 1609 standards [2]. Due to the high mobility and distributed characteristics of VANETS, the MAC layer access protocol has a significant impact on network performance. The MAC protocol in WAVE is described in IEEE 802.11p and IEEE 1609.4 standards. The distributed coordination function (DCF) and the enhanced distributed channel access (EDCA) mechanisms are designed to coordinate the contention-based channel access between vehicles. In DCF or EDCA, the size of the contention window (CW) plays an important role in the access process [3]. However, the current CW adjustment strategy in the WAVE standard is not optimal.

At present, the WAVE and C-V2X standards do not provide a protocol for multi-hop communication between vehicles. Although there are many multi-hop routing protocols in mobile ad hoc networks (MANET), such as DSDV, DSR, and AODV [4,5]. These protocols are not fully applicable in VANETs, because the high mobility of vehicles will cause frequent changes in the network topology, making routing establishment and maintenance difficult, and increasing routing overhead. In recent years, many studies have proposed multi-hop transmission protocols suitable for inter-vehicle communication, but there is still room for improvement in throughput, end-to-end delay, and reliability. For the transmission of emergency messages, multi-hop broadcast communication is considered to be an effective way of inter-vehicle communication. Because the dissemination object of emergency messages is usually not a specific node, but to notify all nodes within a certain range.

The contribution of this paper includes several aspects. First, we propose to use Q-learning and the number of two-hop neighbors to obtain the near-optimal value of the MAC layer CW. A new method for calculating the number of two-hop neighbors under highway scenarios is also designed by using beacon messages and the neighbor tables. To meet the demand for ACK messages in the broadcast communication scenario, we propose a scheme for selecting reply nodes. Finally, a multi-hop communication protocol for broadcast Emergency messages in highway scenarios is proposed based on the MAC layer CW adjustment scheme.

The rest of the paper is organized as follows. Section 2 discusses the related work of the MAC layer contention-based protocols and the multi-hop broadcast protocols in VANETs. The system model with both the beacon message and the emergency message transmission is briefly introduced in Section 3. In Section 4, we propose a Q-learning MAC protocol based on two-hop neighbor detection (QMAC-2ND). A multi-hop broadcast protocol combined with CW adjustment (MBPCA) is proposed in Section 5. Simulation results are shown and analyzed in Section 6. Finally, the conclusion and future work are discussed in Section 7.

## 2. Related Work

### 2.1. MAC Layer Protocol

The DCF and EDCA mechanisms in the WAVE standard use a binary exponential backoff algorithm to adjust the MAC layer CW for unicast communication. They set the initial CW value to *CW_min_*, and increase it exponentially after each transmission failure until it reaches *CW_max_* [6]. In [6,7,8], DCF and EDCA are modeled and simulated, and the CW value is also discussed. The results show that the CW value greatly influences communication performance such as throughput, fairness, and collision probability.

Kloiber et al. in [9] pointed out that the main reason for packet collisions in dedicated short range communications (DSRC/WAVE) is that neighboring nodes select the same backoff counter, and proposed increasing the value of CW to reduce collisions. They also proposed a method called geo-backoff to adjust the backoff window using vehicle geographic location information. A joint scheme of adjusting node transmission power according to vehicle density and adjusting CW according to instantaneous collision rate is proposed in [10]. Amuru et al. [11] pointed out that the exponential back-off mechanism in WAVE is sub-optimal in throughput performance, especially in an unknown dynamic network environment. They modeled the RTS-CTS handshake mechanism as a Markov decision process, and used a post-decision state (PDS)-based learning algorithm to select the backoff window value according to the system state. References [12,13] proposed two methods for adjusting CW based on Q-Learning algorithm with different reward mechanisms. In [10,11,12,13], simulation experiments were performed on their proposed improvement schemes and the WAVE standard scheme or a fixed CW value scheme. The results show that their schemes have improved different communication performances.

However, most of the schemes they proposed cannot cope well with the influence of the change of the number of contention nodes on the CW value. The number of contention nodes significantly impacts on CW value [14,15]. When the number of contention nodes is large, a larger CW can reduce packet collision. On the other hand, using a smaller CW can reduce the transmission delay when the number of contention nodes is small. Another cause of packet collision in VANETS broadcast communication is the existence of hidden nodes. In some studies, the RTS-CTS handshake mechanism is used to solve the problems introduced by hidden nodes, for example [11]. The sending node sends the RTS signal before transmitting the packet, and transmits the packet after receiving the CTS signal from the receiving node. Other surrounding nodes will remain silent when receiving CTS signals to prevent interference. However, the RTS-CTS handshake process will increase the transmission delay. To solve the collision problem of broadcast messages in VANETs, this paper proposes a Q-learning MAC protocol based on detecting the number of two-hop neighbors. Using the number of two-hop neighbors instead of one-hop neighbors can avoid the influence of hidden nodes well.

### 2.2. Multi-Hop Broadcast Protocol

Multi-hop broadcast communication is typically used to transmit Emergency messages in VANETs. Existing multi-hop broadcast protocols can be divided into two categories: sender-based forwarding protocols and receiver-based forwarding protocols. In sender-based forwarding protocols, the current forwarding node selects a node from its neighbor nodes as the next hop forwarding node according to specific rules. In [16], a position-based broadcast mechanism (P-BM) suitable for highway scenes is proposed, which selects nodes that are farther away from the source node and in the lane closer to the source node as the next forwarding node. To limit the redundancy of the message, the forwarding will be stopped when the forwarding range is exceeded. References [17,18] use black burst and binary partition methods to select the node farthest from the source node as the forwarding node. The robust and fast forwarding (ROFF) in [19] uses a neighbor table and an empty space distribution (ESD) bitmap to assign forwarder candidates and assign different waiting delays to each candidate. In [20], a bi-directional stable communication (BDSC) multi-hop broadcast scheme is proposed, which uses beacon messages to estimate the link quality of neighbors and assigns forwarding sequence and forwarding delay to candidate forwarders according to link quality. Wu et al. [21] propose a joint fuzzy relays and network coding-based forwarding (FUZZBR-NC) protocol, which uses a fuzzy logic algorithm to select the next-hop relay node based on the distance, mobility, and RSSI information of the neighbor nodes. FUZZBR-NC selects two forwarding nodes each time to forward packets simultaneously, and uses network coding to improve the packet dissemination ratio.

Receiver-based forwarding includes probabilistic forwarding and delayed forwarding. This category of forwarding protocols usually uses distance and other information to set different forwarding delays and forwarding probabilities for each candidate forwarder. In [22], three probabilistic and timer-based suppression broadcast schemes are proposed: weighted p-persistence, slotted 1-persistence, and slotted p-persistence. Weighted p-persistence assigns greater forwarding probability to farther nodes, slotted 1-persistence assigns shorter forwarding delay to more distant nodes, and slotted p-persistence combines the above two methods. Yang et al. [23] assigns smaller forwarding delay to farther nodes and proposes a location-based adaptive broadcast protocol (PAB) that uses the location, direction, and speed of the vehicle to calculate the forwarding delay. In [24,25], the neighboring vehicles are divided into multiple grids according to their distances, and the farther grids use a smaller forwarding delay. In addition to distance, the protocol proposed in [25] also uses RSSI, speed, and priority to assign different forwarding delays to candidate forwarders. It uses the RTC/CTS handshake mechanism to solve the problems introduced by hidden nodes. Abbasi et al. [26] pointed out that the RTC/CTS handshake mechanism will increase communication overheads. In their intelligent forwarding protocol, the handshake mechanism is removed, and different MAC layer CW values are set for forwarding nodes based on their distance and signal-to-noise ratio. Reference [27] proposed assigning different forwarding probabilities to nodes using an index number, determined by the distance and number of vehicles to adapt to forwarding requirements under different vehicle densities.

Sender-based forwarding saves extra waiting time but results in a lower packet delivery ratio due to packet collisions. Receiver-based forwarding can achieve a higher packet delivery ratio, but the additional waiting time will increase the transmission delay. Zhang et al. [28] combined the sender-based and the receiver-based forwarding schemes. They use their proposed link model to select the preferred forwarder, and the other vehicles will start the forwarding procedure after the waiting timer expires.

This paper proposes some solutions to the problems in single-hop and multi-hop broadcast communication in highway scenarios. For single-hop broadcast, we use Q-learning to train the near-optimal CW values under different vehicle densities through Beacon messages to reduce the collision probability of broadcast packets. By calculating the number of two-hop neighbors, the impact of hidden terminals is reduced without increasing communication overhead. For multi-hop broadcast, a high packet delivery ratio and low transmission delay are achieved by combining sender-based and receiver-based forwarding schemes. The forwarding wait timer is replaced by setting different backoff window values of the MAC layer for forwarding nodes. In this way, the CW obtained by QMAC-2ND trained by beacon messages can simultaneously be applied to the multi-hop broadcast of emergency messages. The emergency messages and beacon messages can participate in the contention-based access process together according to different priorities.

## 3. System Model

This section describes the general V2V network communication model for highway scenarios, including the communication of beacon messages and emergency messages. Moreover, as the fundamental condition of the protocol proposed in this paper, this section introduces the structure of the modified beacon messages and emergency messages.

The paper focuses on the V2V communication system with both beacon and emergency message transmission in highway scenarios. In V2V communication, beacon messages broadcasted periodically between vehicles are widely used in the sharing of vehicle state information. In addition to its function as a common hello message, beacon messages usually include information such as the GPS position, driving direction, speed, acceleration and deceleration, lane change, overtaking, and so on. Sharing this information with nearby vehicles provides vital decision-making assistance for the driving assistance system and unmanned vehicles.

**Figure 1:**
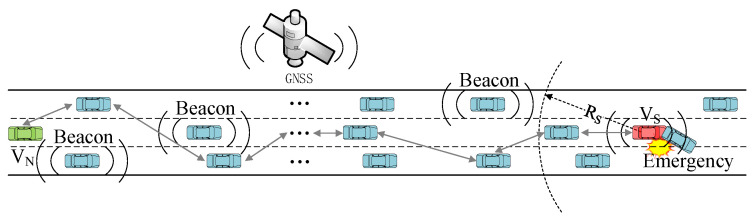
Transmission of beacon and emergency messages in highway scenarios.

The transmission of emergency messages requires multi-hop broadcasting, while the transmission of beacons requires only single-hop broadcasting. Emergency messages are used for the dissemination of emergencies, such as traffic accidents, broken down vehicles, dangerous road conditions, and so on. Beacon messages are not adequate for broadcasting emergency information as their transmission range is very limited. Emergency messages usually require multi-hop communication to notify vehicles at a longer distance, especially on highways where the base station signal is not easy to cover fully. For example, in Figure 1, the vehicle vs. needs to notify all vehicles between the vs. and the V_N_ after a traffic accident. The stability of multi-hop communication is further challenged by the simultaneous transmission of beacon messages during multi-hop transmission of Emergency messages. This paper uses the neighbor state information obtained from the beacon messages to jointly optimize the MAC protocol and the multi-hop broadcast protocol of emergency messages.

Table 1 and Table 2 show the partial structures of the Beacon messages and the Emergency messages, respectively. The *ReplyAddress* is used to indicate the replying node for Beacon broadcast communication. The *MsgID* is the unique ID of each message. *SenderPosition*, *SenderSpeed* and *SenderDirection* are the driving status information of the sending node. *ForwardNeiNum* and *BackwordNeiNum* respectively represent the number of forward and backward neighbor nodes of the sending node, and are used to calculate the number of two-hop neighbor nodes, which will be introduced in Section 4. *ForwarderAddress* is the address of the next-hop forward node in the Emergency message multi-hop broadcast communication. *OriginalPosition*, *TransmitDistance* and *TransmitDirection* determine the transmission range of emergency messages. *Forwardinghop* is the number of hops by which emergency messages are forwarded.

**Figure 2:**
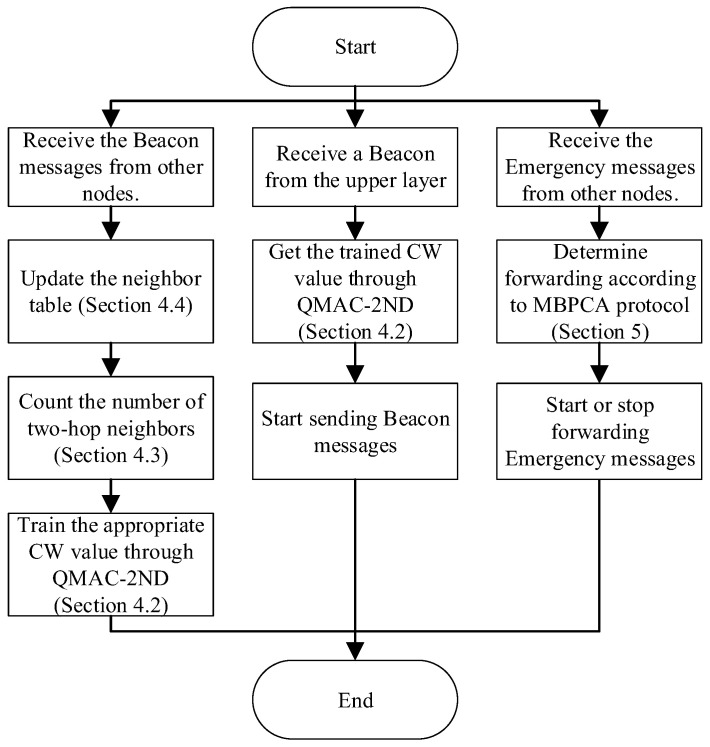
The system flow of the work proposed in the paper.

Figure 2 shows the system flow of the work proposed in this paper. The node uses the beacon messages received from other nodes to update the neighbor table, calculate the number of two-hop neighbors, and train the appropriate CW value. When receiving a beacon message sending request from the upper layer, the node uses the trained MAC layer CW value to send it. For emergency messages, the node determines whether to forward and the waiting time for forwarding according to the MBPCA protocol.

## 4. Contention Window Adjustment in the MAC Protocol

The adjustment of the MAC layer CW size can effectively avoid data collision in V2V communication. This section introduces the CW size adjustment scheme in the WAVE MAC protocol and proposes a Q-Learning MAC protocol based on two-hop neighbor detection.

**Figure 3:**
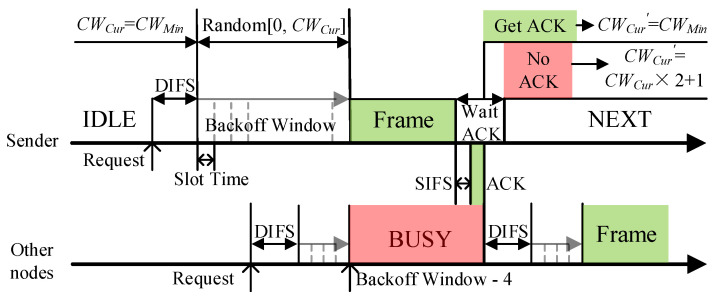
The contention-based MAC protocol process of the WAVE standard.

DCF in the WAVE standard is a distributed access algorithm using carrier sense multiple access with a collision avoid (CSMA/CA) mechanism in each node, which allows a node to obtain sending opportunities by contending channel. The contention process is shown in Figure 3. Before sending a frame, the node will detect the channel state and execute the backoff window after the channel has been idle for DIFS (DCF interframe space) time. The value of the backoff window is a random integer from [0, *CW_Cur_*], where *CW_Cur_* is the current CW value. The backoff window is equivalent to a timer, and data frames can only be sent when the value of the backoff window decreases to 0 in units of time slots. If the channel becomes busy before the backoff window is reduced to 0, it will lock the current value and continue execution when the channel is idle again.

Predictably, the value of CW is crucial in the access process. When the vehicle density is high, a small CW will cause many data collisions. However, too large a CW will lead to increased communication delay. In WAVE, the binary exponential backoff algorithm is used to adjust the CW. The initial value of CW is *CW_Min_*. After each transmission failure or no confirmation message (ACK) is received, the CW value is increased to *CW_Cur_* × 2 + 1 until it reaches *CW_Max_*. And CW will be reset to *CW_Min_* after successful transmission. The ACK message is sent by the receiver after receiving the packet and passing the short interframe space (SIFS) time.

The EDCA algorithm defines four access categories at the MAC layer, represented as AC[0]–AC[3], to meet the different QoS requirements for various services. The priority is divided by assigning different contention parameters to each access category, with AC[3] having the highest priority. EDCA uses AIFS [AC] instead of DIFS in DCF, and access categories with higher priority have smaller arbitration inter frame space (AIFS). Meanwhile, the frames with different priorities from the application layer are stored in four queue buffers, and the frame contention process in different queues is similar to the contention process between different nodes. That is, one node is divided into four virtual nodes with different priorities.

Since both DCF and EDCA contention processes rely on ACK messages, one of the neighbor nodes will be selected to reply the broadcast message. The selection scheme of the replying nodes will be introduced in detail later.

### 4.1. The Q-Learning Algorithm

Q-learning is a classic algorithm in reinforcement learning, which is used in model-free learning. It is suitable for application in VANETs because it can interact with the environment and consume less computing power. When used in VANETs, each vehicle node in the network can be regarded as an independent agent. The agent acquires the current environment state and decides the next action to take based on the learning experience. After the action acts on the environment, a reward and the next state will be feedback to the agent. Through this cyclical interaction between the agent and the environment, the agent can gradually generate predictions of rewards or punishments from past learning experiences, and perform the action that can obtain the maximum rewards.

The triples (*S*, *A*, *R*) are used to model the Q-learning process, in which, *S* is the set of all possible states of the agent, *A* is the set of possible agent actions under state *s* ∈ *S*, *R* is the reward for transitioning from state *s* to state *s’*, by taking action *a* ∈ *A*. At each discrete time *t*, the agent selects the next action *a* to execute according to the current state *s_t_* and its own Q-table, and then obtains the reward value *R* according to the reward function. The Q-table consists of *S’* rows and *A’* columns, with the elements *Q* (*s*, *a*), where *S’* and *A’* are the sizes of set *S* and set *A* respectively, and *Q* (*s*, *a*) is given by:

(1) $$Q(s_{t},a_{t})\leftarrow Q(s_{t},a_{t})+\alpha \times [r_{t}+\gamma \times \max_{a_{t+1}}Q(s_{t+1},a_{t+1})-Q(s_{t},a_{t})].$$

During the Q-learning process, the Q-table is continuously updated according to Equation (1). The discount factor *γ* ∈ [0, 1] is used to indicate the importance of future rewards relative to immediate rewards. The larger the value, the more important the future rewards. *γ* = 0 means that only current rewards are considered, in which case the agent is short-sighted. *α* ∈ [0, 1] is the learning rate, indicating the proportion of newly acquired information used for learning. *α* = 0 means that the agent will no longer learn any new information.

### 4.2. QMAC-2ND Design

**Figure 4:**
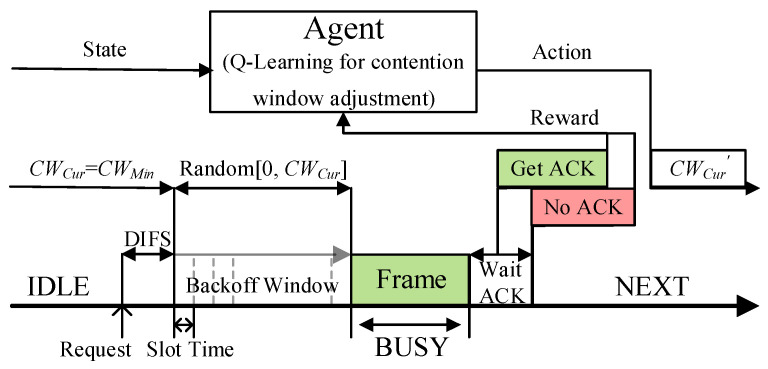
The contention process of the QMAC-2ND.

QMAC-2ND uses the Q-learning algorithm to train the near-optimal CW value under different vehicle densities to reduce communication collisions in broadcast communications. As shown in Figure 4, each vehicle node is regarded as an independent agent, the number of two-hop neighbors is used as the state space, the different CW values are used as the action space, and whether the ACK message from the replying node is received is used as the judgment criterion of the reward. When a node needs to transmit a message, it will judge the current state based on the number of two-hop neighbors at this time, and adopt the action (that is, CW value) with the largest accumulated reward value in the current state. Since the algorithm proposed in this paper does not use deep reinforcement learning, very little computing power is required from the agent.

Due to the fast movement of vehicles, the communication environment of VANETs is characterized by rapid changes. The different number of communication nodes directly affects the selection of the best CW value. According to the number of two-hop neighbor nodes, the state space is divided into four states: [0, 10], [11, 30], [31, 70], and [71+]. The division of the state space is related to the effective communication range of the nodes and determines whether the appropriate CW value can be obtained under different vehicle densities. Here, it is divided according to the experimental results obtained using different CW values under different numbers of nodes. The calculation method of the number of two-hop neighbor nodes is introduced in Section 4.3 below.

The action space, set according to the CW value used by the security messages in EDCA, is [3, 7, 15, 31, 63, 127, 255]. Based on the four possible states and seven possible actions, the size of the Q-table maintained by a node is 4 × 7. The appropriate size of the Q-table can effectively improve the convergence speed and reduce the consumption of computing resources and time. To balance the exploration and exploitation process, Q-learning usually selects the next action to be performed using the ε-greedy strategy. In the exploration process, an action is selected at random in the action space with probability *ε*, so that it is possible to try any action in any state. In contrast, with probability 1 − *ε*, the process of exploitation selects the optimal policy *π* in the current state *s* as the next action according to the Q-table. *π*(s) is given by Equation (2), representing the action with the largest Q value in the current state *s*.

(2) $$\pi (s)=\underset{a}{\mathrm{argmax}}Q(s,a).$$

*Q*(*s*, *a*) in the Q-table is initialized to 0 at the beginning of the learning process, at which point the agent needs more time to execute the exploration process to traverse all states. Therefore, setting *ε* to a bigger value at the beginning of the learning process can increase the convergence speed. After convergence, the agent performs the exploitation process more based on the learned knowledge. In this paper, *ε* decreases linearly with time according to Equation (3):

(3) $$\varepsilon (s)=\left\{ \begin{array}{l} 1-T_{Trained}(s)/T_{Set},\varepsilon >0.05\\ 0.05,\;\varepsilon \leq 0.05 \end{array}\right.,$$

where *T_Set_* is the preset training time, *T_Trained_*(*s*) is the current training time under different states *s*. When the number of neighbors is at a fixed level *s*, *ε* decreases linearly with time until it reaches the preset minimum value of 0.05. Since the state *s* changes according to the number of neighbor nodes, independent *ε* values are assigned to the four states to ensure the fastest convergence in different vehicle density environments. For example, when the number of two-hop neighbors is [0, 10], the agent converges when *ε* has decayed to the minimum value of 0.05, but when the number of two-hop neighbors is changed to [11, 30], the agent still needs a larger *ε* for the exploration process. Setting the minimum *ε* to 0.05 ensures that even after the agent reaches the convergence state, it still spends 5% of its time in the exploration process to correct the learned experience continuously.

The reward function also determines the convergence speed and the degree of reinforcement learning. According to [12,13], when the agent receives an ACK message, it gains a positive reward, otherwise it receives a negative punishment of −1. At the same time, considering that a large CW value will cause a large transmission delay, the reward value is divided into [1, 6/7, 5/7, 4/7, 3/7, 2/7, 1/7] according to different CW. Using a smaller CW will receive a larger reward value after the transmission is successful; this makes the agent prefer the smallest CW that ensures successful message transmission. However, if the difference between the reward values is too significant, the agent will only focus on the delay sensitivity and ignore the impact of the ACK message. Instead of [1, 6/7, 5/7, 4/7, 3/7, 2/7, 1/7] in [12], the proposed reward function sets the reward values to [1, 0.95, 0.9, 0.85, 0.8, 0.75, 0.7] based on the experimental data, which can make the agent take into account the delay sensitivity and the transmission success ratio at the same time. Although ACK messages are only used in unicast in the WAVE standard, we extend the use of ACK messages for broadcast communications in Section 4.4 below.

### 4.3. Calculation of the Number of Two-Hop Neighbors

**Figure 5:**
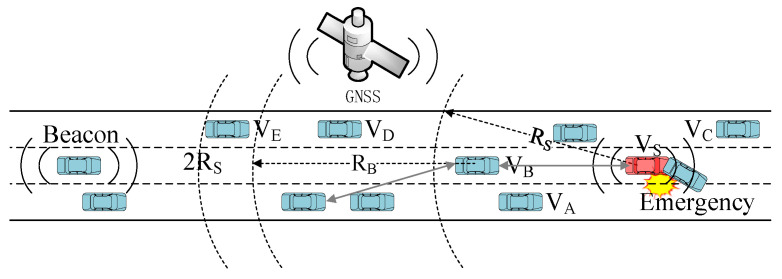
Calculation of the number of two-hop neighbors in the highway scenario.

Since hidden terminals can also cause collisions on data transmission, they should be taken into consideration in the research of MAC protocols. For example, in Figure 5, when node vs. sends a data packet to node V_B_, it may collide with the data packet from hidden node V_D_, causing the transmission to fail. Since V_D_ is not within the communication range of V_S_, so it is hidden to V_S_, and vs. cannot avoid collision with data packets from V_D_ by monitoring the state of the channel.

The MAC protocols based on vehicle density (or the number of neighbors) can avoid transmission collisions by estimating near-optimal CW values. However, the calculation of vehicle density is mainly based on large-scale traffic flow estimation or estimation based on historical data, which makes it challenging to obtain the accurate vehicle density in the local area near the sending node. The number of one-hop neighbors within the communication radius of the sending node can be easily obtained through the periodic broadcast of beacon messages. But that does not eliminate the influence of hidden neighbors. Some researches propose to estimate vehicle density based on the number of received beacon messages, but the accuracy is usually low. Reference [29] suggests adding the IDs of all its neighbor nodes to the beacon message, and the receiving node obtains the number of two-hop neighbor nodes by retrieving the number of non-common neighbors. This method can accurately obtain the number of two-hop neighbors to design the MAC protocol better to avoid the influence of hidden neighbors. But it will increase the overhead of beacon messages, especially when the number of neighbors is large.

This paper presents a new method for calculating the number of two-hop neighbors by using Beacon messages and GPS information of neighbor nodes. Each node maintains a local neighbor node table according to the received Beacon messages. The neighbor table contains the ID and GPS location information for each neighbor. Based on the location of its one-hop neighbors, the node can calculate the number of neighbors in its forward direction and backward direction, respectively. The numbers of forward-neighbor and backward-neighbor nodes are added to the beacon message with only a small overhead. In the paper, “neighbor” refers to “one-hop neighbor”.

**Figure 6:**
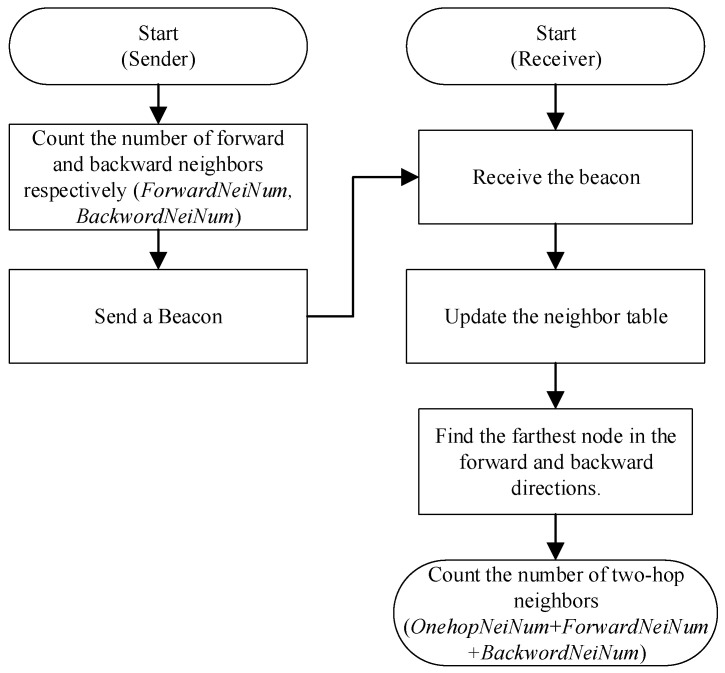
Two-hop neighbors number calculation.

Figure 6 shows the process of calculating the number of two-hop neighbors. The sender periodically broadcasts beacon messages, which include the number of forward-neighbors and backward-neighbors of the sending node. Upon receiving a beacon message, the receiver updates its local neighbor table, which includes the number of forward and backward neighbors for each neighbor. The structure of the local neighbor table and its updating principle are described in the next subsection. We obtain the farthest neighbors in the forward and backward directions by looking up the neighbor table. Then we calculate the number of two-hop neighbors by adding the number of forward neighbors of its farthest forward neighbor (*ForwardNeiNum*), the number of backward neighbors of its farthest backward neighbor (*BackwordNeiNum*), and the number of its own one-hop neighbors (*OnehopNeiNum*). *OnehopNeiNum* is equal to the size of the neighbor table.

Note that the number of two-hop neighbors is not equal to the number of nodes within twice the propagation range of the source node. For example, the V_E_ in Figure 5 is in the 2*R_S_* range, but it is outside the communication range of V_B_, the farthest neighbor of V_S_, so it is not a two-hop neighbor of V_S_. And V_E_ will not interfere with the communication between vs. and V_B_. Our proposed method for calculating the number of two-hop neighbors is currently only applicable to highway scenarios. The calculation of two-hop neighbors in urban scenes needs to be further researched according to the urban road structure.

### 4.4. ACK Message in Broadcast Communication

**Figure 7:**
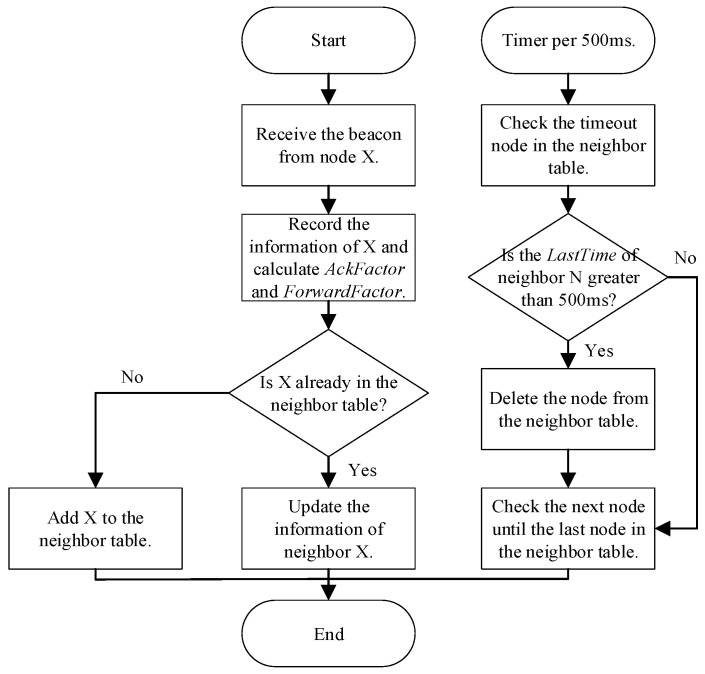
The neighbor table update process.

According to the neighbor table, one of the neighbors is selected as the response node to apply the ACK message to the broadcast communication scenario. The neighbor tables are maintained and updated using information from received beacon messages. Table 3 shows the structure of the neighbor table, where *RSSI* is the received signal strength indication, *LastTime* is the time when the beacon sent by that neighbor was last received, *AckFactor* is the metric used to select reply node for the beacon, and *ForwardFactor* is the metric used to select forwarding node in multi-hop broadcast communication. The neighbor table update process is shown in Figure 7.

The node selects the neighbor with the largest *AckFactor* from the neighbor table as the replying node of the received Beacon. *AckFactor* is calculated based on the distance, speed, driving direction, and RSSI of the neighbor nodes, as shown in Equation (8), where *a*, *b*, *c*, *d* are weight factors, indicating the importance of each factor, and a+b+c+d=1. The distance factor *DF*(*x*), the direction factor *DI*(*x*), the mobility factor *MF*(*x*), and the RSSI factor *RF*(*x*) are given by Equations (4)–(7) respectively, where *d*(*x*) is the distance between the *x*-th neighbor and the sending node, *R* is the reference transmission radius, vs. is the speed of the sending node, *V*(*x*) is the speed of the *x*-th neighbor, *RSSI*(*x*) is the *RSSI* of the latest beacon received from the *x*-th neighbor, and *RXThresh* is the receiving sensitivity of the physical layer. In practical applications, the effective transmission radius of each node is not fixed but is determined by multiple factors such as transmitting power, antenna gain, and physical channel environment. Therefore, *R* here is only used as an approximate reference value. When the actual transmission distance is greater than *R*, *DF*(*x*) is equal to 0. In addition, *a* = 0.5, *b* = 0.1, *c* = 0.2, *d* = 0.2 in this paper. *ACKFactor* is used to select a neighbor a short distance away that travels in the same direction, has a small speed difference with itself, and has a large RSSI as the replying node.

(4) $$DF(x)=\left\{ \begin{array}{l} (R-d(x))/R,d(x)<R\\ 0,d(x)\geq R \end{array}\right.,$$

(5) $$DI(x)=\left\{ \begin{array}{l} 1,Same\;direction\\ 0,Opposite\;direction \end{array}\right.,$$

(6) $$MF(x)=\left\{ \begin{array}{l} 1-|(V_{S}-V(x))/V_{S}|,MF(x)>0\\ 0,MF(x)\leq 0 \end{array}\right.,$$

(7) RF(x)=|RSSI(x)/RXThresh−1|,

(8) AckFactor(x)=aDF(x)+bDI(x)+cMF(x)+dRF(x),

### 4.5. Detailed Implementation of QMAC-2ND Algorithm

Algorithm 1 describes the detailed implementation of QMAC-2ND, where *s_t_* ∈ [0, 3] represents four different levels of the number of two-hop neighbors, 0, 1, 2, 3 correspond to [0, 10], [11, 30], [31, 70], and [71+], respectively. And *a_t_* ∈ [0, 6] represents seven different CWs in *Action*[7]. When the ACK times out, the *AckFactor* of the replying node in the neighbor table is set to 0. This feedback mechanism ensures that when the ACK of the current replying node times out, it will not be selected as the replying node next time, and its *AckFactor* will not be updated again until the beacon message from the node is received again.
**Algorithm 1: QMAC-2ND**1: **Initialize** *Q*(*s_t_*, *a_t_*) = 0, *s_t_ =* 0, *a_t_* = 0, *CW_t_* = 0, *Action*[7] = {3, 7, 15, 31, 63, 127, 255},     *Reward* = [1, 0.95, 0.9, 0.85, 0.8, 0.75, 0.7], at *t* = 0;2: **repeat for each episode**3:       **procedure** Update_Environment()4:           Get the number of two-hop neighbors $N_{Nei}^{2}$;5:           Update *s_t_* based $N_{Nei}^{2}$;6:           **if** *T_Trained_*(*s*) < *T_Set_* and *ε* > 0.057:               Update *α*, *ε* according to Equation (3);8:           **else**9:               *α* = *ε* = 0.05;10:         **end if**11:     **end procedure**12:     Choose_Action() *a_t_* according to ε-greedy;13:     Choose_ReplyNode() *N_x_ according to AckFactor*(*x*);14:     Send packets using *CW_t_* = *Action*[*a_t_*];15:     **procedure** Wait_ACK()16:         **if** get ACK message17:             *r_t_* = *Reward*[*a_t_*];18:         **else**19:             *r_t_* = −1; *AckFactor*(*x*) = 0;20:         **end if**21:     **end procedure**22:     Update *Q-table* according to Equation (1);23:     **Next** episode;24: **until** *s* is terminal;

## 5. Multi-Hop Broadcast Protocol Combined with Contention Window (CW) Adjustment

The multi-hop broadcast communication protocol focuses on the forwarding process, including selecting the forwarding nodes and setting the forwarding delays. This section introduces a forwarding scheme that combines sender-based and receiver-based forwarding and utilizes the MAC layer CW adjustment scheme to determine the forwarding delay.

### 5.1. The Preferred Forwarder Selection Scheme

Selecting a node from the neighbor table by the previous sender as the next forwarding node can save forwarding time. As with the selection scheme of the answering node of the Beacon message, we use the distance, driving direction, speed, and RSSI of the neighbors as the reference for selecting the next forwarding node. The difference is that the *DF*(*x*) is modified to choose more distant neighbors, as shown in Equation (9). *ForwardFactor* is given by Equation (10), and the neighbor with the largest *ForwardFactor* will be the preferred forwarder.

(9) $$DF^{\prime }(x)=\left\{ \begin{array}{l} d(x)/R,d(x)<R\\ 1,d(x)\geq R \end{array}\right.,$$

(10) $$ForwardFactor(x)=aDF^{\prime }(x)+bDI(x)+cMF(x)+dRF(x).$$

Instead of the timeout timer, we use the backoff window of the MAC layer protocol as the forwarding delay. In this way, the node can better participate in the contention process of the MAC layer, especially in the presence of beacon message propagation. In our proposed multi-hop broadcast protocol, the backoff window takes a random integer between [*ForwardCW_min_*, *ForwardCW_max_*] instead of between [0, *CW_Cur_*] as in the WAVE standard. The preferred forwarder selects a random integer from [0, *ForwardCW_max_*] as the backoff window for forwarding the emergency message. *ForwardCW_max_* is given by Equation (11), where *CW*(*x*) is the near-optimal value of CW obtained from the QMAC-2ND algorithm, and *d_min_* is the distance between the forwarding node and its nearest neighbor in the opposite direction of the message transmission direction. The transmission direction of the emergency message is determined by the direction of the road and is given by *TransmitDirection* in the message. For example, the vs. in Figure 5 transmits an Emergency message backwards. V_B_ is the preferred forwarder, then *d_min_* is the distance between V_B_ and V_A_. *d_min_* can be calculated from the neighbor table.

(11) $$\begin{array}{cc} {ForwardCW}_{max}(x) & =\left\lceil \left(1-\left(R-d_{min}(x)\right)/R)\times CW(x\right)\right\rceil \\ & =\left\lceil \left(d_{min}(x\right)/R)\times CW(x)\right\rceil \end{array}$$

### 5.2. CW of the Candidate Forwarder

Receiver-based delayed forwarding increases transmission latency, but a large number of candidates can increase the transmission success rate. Delayed forwarding usually saves more time than retransmission. The value of the backoff window of the candidate forwarder is a random integer selected from [*ForwardCW_min_*, *ForwardCW’_max_*], *ForwardCW_min_* and *ForwardCW’_max_* are given by Equations (12) and (13) respectively.

(12) $${ForwardCW}_{min}(x)=\left\lceil \left(1-d(x)/R)\times CW(x\right)\right\rceil ,$$

(13) $$ForwardC{W^{\prime }}_{max}^{}(x)=\left\lceil \left(1-\left(d(x)-d_{min}(x)\right)/R)\times CW(x\right)\right\rceil .$$

**Figure 8:**
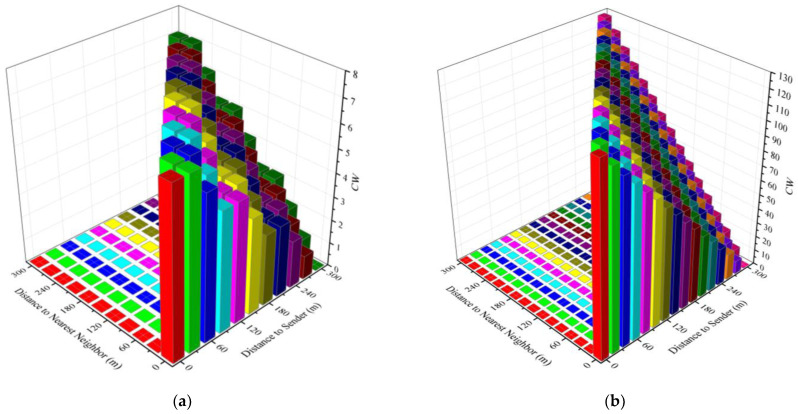
The *ForwardCW_min_* and *ForwardCW_max_* values when CW = 7 and CW = 128, respectively. (**a**) The *ForwardCW_min_* and *ForwardCW_max_* values with different *d*(*x*) and *d_min_*(*x*) when CW = 7; (**b**) the *ForwardCW_min_* and *ForwardCW_max_* values with different *d*(*x*) and *d_min_*(*x*) when CW = 128.

It can be seen that the final backoff window value used by the candidate forwarder is most likely smaller than that of the preferred forwarder unless the distance of the candidate forwarders is greater than *R* and *ForwardCW’_max_* < *ForwardCW_max_*. The use of *d_min_* can make the backoff window of the forwarder different from that of its neighbors. This division method is similar to dividing the road into a fixed number of grids in [24,25], and each grid uses a different delay to forward. However, unlike the grid method, variable numbers of grids can be divided according to different values of *d_min_*(x) and *CW*(*x*). The variable number of grids allows more fine-grained partition of forwarding delays to avoid forwarding collisions between neighbors. Assume *R* = 300 m, Figure 8a,b show *ForwardCW_min_* and *ForwardCW_max_* when *CW(x)* is 7 and 128, respectively. In the figures, *ForwardCW_min_* is the value corresponding to different *d*(*x*) (distance to sender) when *d_min_*(*x*) = 0, and *ForwardCW_max_* is the value corresponding to different *d_min_*(*x*) (distance to nearest neighbor) when *d*(*x*) is constant. For example, when the candidate forwarder is 300 m away from the previous forwarding node and 60m from the nearest candidate forwarder, and *CW*(*x*) = 128, its *ForwardCW_min_* = 0 and *ForwardCW_max_* = 26.

### 5.3. Detailed Implementation of the Multi-Hop Broadcast Protocol Combined with CW Adjustment (MBPCA)

Algorithm 2 details the implementation of the MBPCA. The *ForwarderAddress* is given in the packet and represents the next preferred forwarder. *CurTransHop* and *MsgHop* are respectively the number of forwarding hops cached by the node and the number of forwarding hops obtained from the message. By contrast with the ACK messages of Beacon messages, the Emergency message forwarded to the next-hop is regarded as the ACK message of the previous forwarding node. If the forwarder receives an ACK while waiting for forwarding, the current forwarding procedure is canceled to reduce the forwarding redundancy of the message. The region of interest is determined by *OriginalPosition*, *TransmitDistance*, and *TransmitDirection* in the packet. Only nodes located in the message transmission direction and within the interest region can be selected as the preferred or candidate forwarders. For example, the V_C_ in Figure 5 cannot be the forwarder for the next hop.

If the received ACK message is not from the preferred forwarder or the ACK times out, the *ForwardFactor* of the preferred forwarder in the neighbor table is set to 0. This feedback mechanism ensures a different neighbor is chosen as the preferred forwarder for the subsequent forwarding or retransmission. The timeout retransmission function was added to improve the success rate of message transmission. In the process of waiting for retransmission, if the next-hop retransmission is received from other neighbors, the current retransmission procedure will be canceled. The retransmission cancelation mechanism, together with the limit on the number of retransmissions, can reduce message redundancy caused by retransmissions.
**Algorithm 2: MBPCA**1: Node *V_x_* receives an Emergency message from *V_s_*;2: **if** *ForwarderAddress == ID*(*x*)      //preferred forwarder3:       **if** duplicate (*MsgID*)4:           **if**
*CurTransHop* < *MsgHop*;5:               **if** wait to retransmission6:                   Cancel retransmission procedure;7:               **end if**8:               **if** wait Ack || wait to forward9:                   Cancel Ack timeout timer or forwarding;10:             **end if**11:         **end if**12:     **else if** within the region of interest13:         *CurTransHop* = *MsgHop*;14:         Calculate the forwarding CW according to Equation (11);15:         Choose preferred forwarder according to Equation (10);16:         Start the MAC layer forwarding procedure and start the Ack timeout timer after send successfully;17:     **end if**18: **else**                //candidate forwarder19:     **if** duplicate (*MsgID*)20:          Follow the procedures in lines 4–11;21:     **else if** within the region of interest22:          *CurTransHop* = *MsgHop*;23:          Calculate the forwarding CW according to Equations (12) and (13);24:          Follow the procedures in lines 15–16;25:     **end if**26: **end if**27: **procedure** AckTimeout()28:     **if** enable retransmission && less than the limit of retransmission times29:          Start the retransmission procedure;30:     **else**31:          Drop the packet;32: **end procedure**

## 6. Performance Evaluation

In this section, the proposed QMAC-2ND and MBPCA are simulated and compared with other related schemes. Several different metrics are used to evaluate the single-hop broadcast of beacon messages and the multi-hop broadcast of emergency messages, respectively.

### 6.1. Simulation Setup

The Veins (Vehicle in Network Simulation) simulation platform [30], which is composed of a road simulator SUMO (Simulation of Urban Mobility) [31] and a discrete event simulator OMNeT++ [32], is used for our simulation experiments. A two-way four-lane highway with a total length of 2.5 km is used to complete the simulation experiment. The vehicle uses the Krauss car-following model to drive at a maximum speed of 38.89 m/s. First, the vehicle training process is completed in a closed-loop road environment with variable vehicle density. Then, the vehicles use the convergent Q-table obtained after training to perform the evaluation process for 300 s when the vehicle density is 5, 10, 30, 50, and 70 vehicles per kilometer, respectively. Vehicle density is controlled by the minimum distance between vehicles, and the maximum speed of vehicles varies with different vehicle densities.

Table 4 shows the main simulation parameters. The transmission range of the vehicle is not fixed but jointly determined by its transmission power, antenna model, and the physical layer channel model to make the simulation closer to reality. The *R* used in our algorithm is only a reference approximation. The actual transmission range of a node may be greater than or less than *R*. To ensure the repeatability and accuracy of the simulation, each group of experiments uses the same random number seed, and the final experimental result is the average of three groups of experimental results with different random number seeds.

### 6.2. Evaluation of QMAC-2ND

The QMAC-2ND protocol is evaluated using single-hop broadcast Beacon message transmission and is compared with the modified-WAVE protocol and the QMAC protocol proposed in [13]. The modification of WAVE is reflected in the use of the replying node selection scheme in the broadcast communication scenario proposed in this paper. This is because the reply scheme of the current WAVE standard is only suitable for unicast communication. In addition, the WAVE standard only changes the CW value on retransmission. To facilitate the evaluation of performance, this paper does not allow beacon message retransmission in the MAC protocol. Therefore, the modified-WAVE protocol uses an exponentially increasing CW value when transmitting the next beacon message after a transmission failure, instead of using it during retransmission. The MAC protocol uses the average packet delivery ratio, the average one-hop delay, and the fairness index as evaluation metrics.

**Figure 9:**
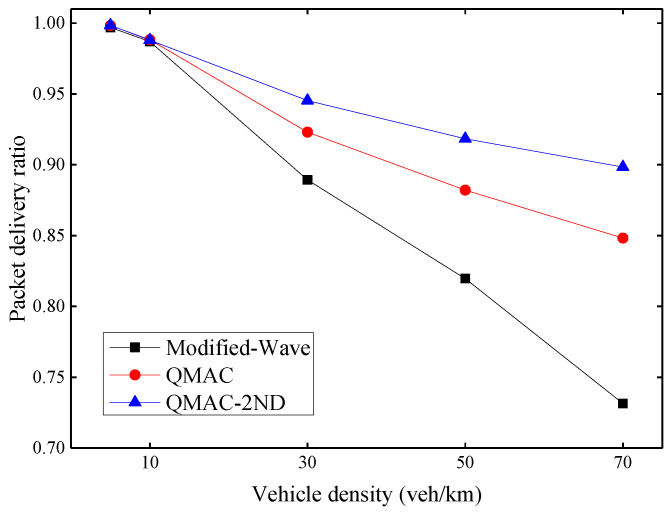
Average packet delivery ratio under different vehicle densities.

Figure 9 shows the average packet delivery ratio under different vehicle densities. The packet delivery ratio here is the ratio of the number of beacon messages received ACK to the total number of beacon messages sent. When the vehicle density is 5 or 10 vehicles per kilometer, all three protocols can achieve a high packet delivery ratio, because data collisions rarely occur when the number of nodes is small. When the vehicle density exceeds 30 vehicles per kilometer, the packet delivery ratio of the modified WAVE drops rapidly. This is because it increases its CW value only after a transmission failure. Both QMAC and QMAC-2ND perform better than the modified-WAVE. With the increase of vehicle density, the performance advantages of QMAC and QMAC-2ND over the modified WAVE protocol also increase. QMAC-2ND performs better than QMAC because it takes into account the number of contention nodes and the influence of hidden nodes. QMAC-2ND improves the packet delivery ratio by up to 16.7% compared with the modified-WAVE, and up to 5.0% compared with QMAC.

**Figure 10:**
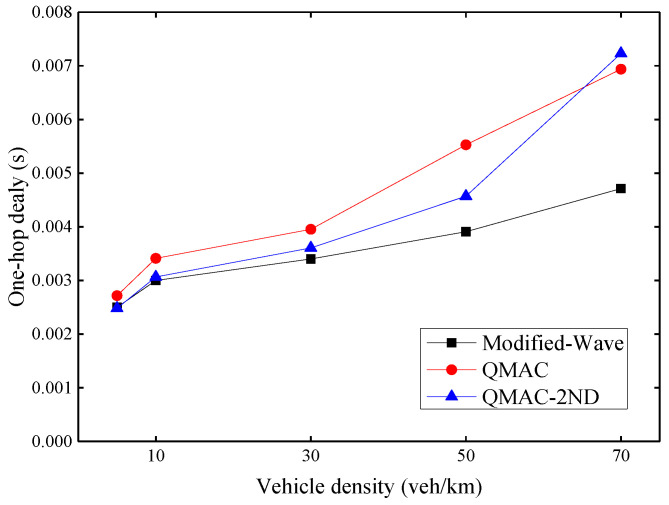
Average one-hop delay under different vehicle densities.

Figure 10 shows the average one-hop delay under different vehicle densities. As the density of vehicles increases, the one-hop delay increases for all three protocols. However, the delay increase trend of the modified-WAVE is slight. This is because the modified-WAVE cannot obtain a higher packet delivery ratio by quickly adjusting the CW value. It will reset the CW value to the minimum after each successful transmission. Therefore, although the modified-WAVE has the lowest delay, it is at the expense of a lower packet delivery ratio. QMAC can increase the packet delivery ratio by using a larger CW value, but the CW value cannot be adjusted quickly according to changes in vehicle density. It is possible that a large CW value is still maintained for some time after the vehicle density has reduced. Our proposed QMAC-2ND protocol can quickly respond to the changing vehicle density, and use the appropriate smallest CW value under the premise of ensuring the packet delivery ratio. When the vehicle density is 70 veh/km, QMAC-2ND has the largest one-hop delay because it sets a larger CW value according to the vehicle density to improve the packet transmission success rate. QMAC has a certain lag in dealing with changes in vehicle density, although its one-hop delay is relatively low, its improvement in packet delivery ratio is poor.

Fairness is an essential metric of MAC layer protocol evaluation to indicate whether each node has the same opportunity to access the channel. We use Jain’s fairness index proposed in [33] shown in Equation (14) as the fairness evaluation metric, where *x_i_* is the throughput of node *i*, and *N* is the number of nodes.

(14) $$FI=\frac{{\left(\sum_{i=1}^{N}x_{i}\right)}^{2}}{N\sum_{i=1}^{N}x_{i}^{2}},$$

**Figure 11:**
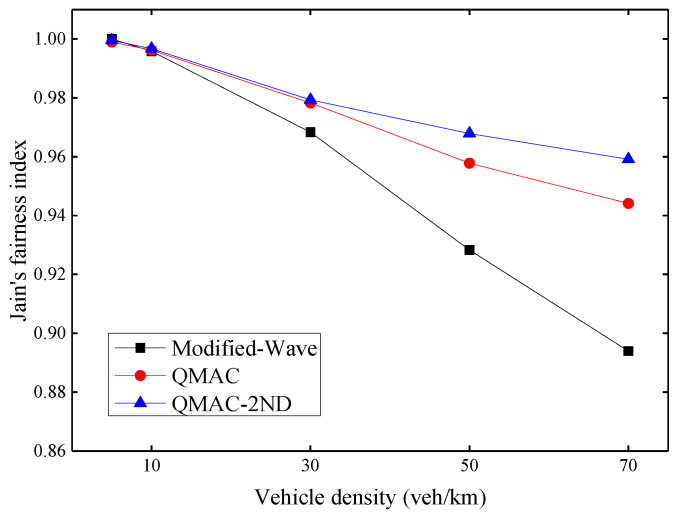
Jain’s fairness index under different vehicle densities.

The fairness index is divided into long-term and short-term indexes. Since the communication of VANETs is mostly security-related messages, it is necessary to ensure that all nodes in the network have the opportunity to access the channel in a short time. Therefore, this paper focuses on short-term fairness, which uses the throughput of nodes per second for statistical calculations. Figure 11 shows Jain’s fairness index under different vehicle densities. As the density of vehicles increases, the fairness problem gradually becomes prominent. QMAC-2ND improves fairness by up to 6.5% compared with the modified-WAVE, and up to 1.5% compared with QMAC.

### 6.3. Evaluation of MBPCA

Our proposed multi-hop broadcast protocol, MBPCA, is evaluated using the transmission of emergency messages and is compared with the flooding broadcast based on the modified-WAVE, the weighted p-persistence broadcast (WPB) in [22], and the PAB in [23]. WPB includes a retransmission mechanism, when a node does not receive the forwarding from the neighbor within the waiting period, it will forward the message with a probability of 1. We separately evaluate MBPCA without retransmission mechanism and MBPCA with one retransmission. The number of retransmissions can be set in the simulation.

For comparative analysis, we use the following metrics:

- Packet delivery ratio: The ratio of the number of emergency messages forwarded to 2 km away or to the end of the road to the total number of emergency messages sent;
- End-to-end delay: The multi-hop forwarding delay between the source node and the last forwarding node;
- Reliability factor: The ratio of the average number of unique emergency messages received by nodes within the range of interest to the total number of emergency messages sent;
- Redundancy factor: The ratio of the average number of repeated emergency messages to the average number of unique emergency messages received by nodes in the interest range.

**Figure 12:**
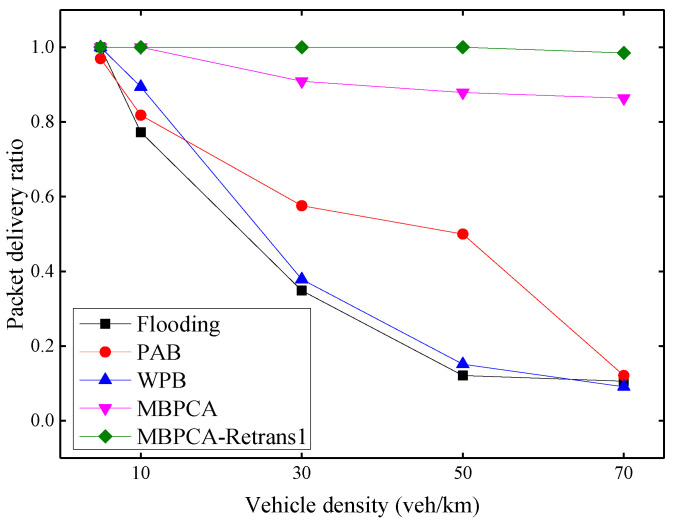
Packet delivery ratio under different vehicle densities.

The packet delivery ratio under different vehicle densities is shown in Figure 12. With the increase of vehicle density, the average packet delivery ratio has a downward trend overall due to the increase in collision probability. When the vehicle density is 70 vehicles per kilometer, the packet delivery ratio of flooding, PAB and WPB drop to only about 1%. Both MBPCA without retransmission and MBPCA with one retransmission have better packet delivery ratios. Even when the vehicle density is 70 vehicles per kilometer, the packet delivery ratio of MBPCA can still reach approximately 86%. MBPCA with one retransmission maintains 100% packet delivery ratio when the vehicle density is 5, 10, 30, and 50 veh/km (vehicles/kilometer). Even when the vehicle density is 70 vehicles per kilometer, its packet delivery ratio still reaches about 98.5%.

**Figure 13:**
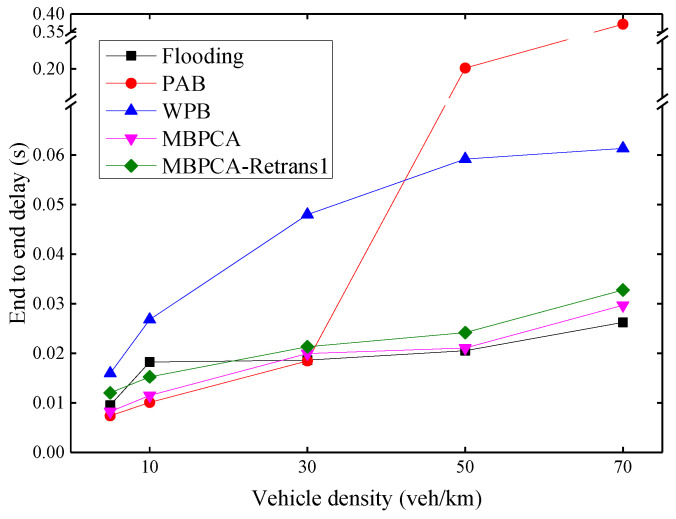
Average end-to-end delay under different vehicle densities.

As shown in Figure 13, the average end-to-end delay increases as the density of vehicles increases. PAB has the worst delay when the vehicle density exceeds 50 veh/km. This is because its forwarding waiting delay is approximately exponentially related to distance. When there is no distant forwarding node or the packet forwarded by a distant forwarding node fails, this will cause a greater delay. WPB also has a high delay, because it has to wait a fixed time, for example, 4 ms, before each forwarding and retransmission. MBPCA has low latency, the longest delay is less than 30 ms, which meets the delay requirements of emergency messages. Although there is a 20 ms waiting time before retransmission, the average end-to-end delay of MBPCA with one retransmission increases very little because only very few packets need to be retransmitted. The flooding protocol performs best in terms of end-to-end delay when the vehicle density exceeds 50 veh/km, but it is at the expense of a packet delivery ratio.

**Figure 14:**
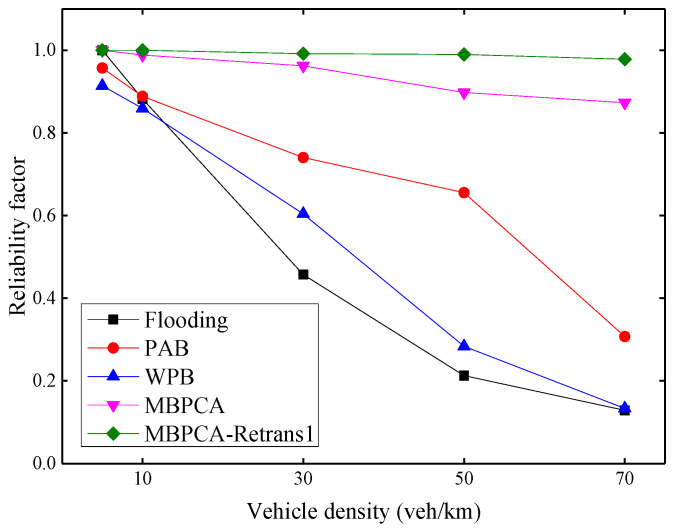
Reliability factor under different vehicle densities.

The reliability factor shown in Figure 14 is used to evaluate the receiving ratio of emergency messages of all nodes in the range of interest. Because the emergency messages not only must be notified to distant vehicles but also all vehicles within a specific region. With the increase of vehicle density, the reliability factors of the flooding, PAB, and WPB protocols decrease obviously. Because beacon messages are periodically transmitted in the network while transmitting emergency messages, an increase in the number of vehicles will increase the number of packets transmitted on the network, thereby increasing the collision probability. Although the reliability factor of MBPCA also shows a slight decrease, it can still maintain very high reliability. When the vehicle density is 70 veh/km, its reliability factor is still approximately 87%. Because we combine the MAC layer contention mechanism in the forwarding scheme, and assign a different backoff window to each candidate forwarding node as much as possible. The MBPCA with one retransmission can always maintain a reliability factor close to 100%.

**Figure 15:**
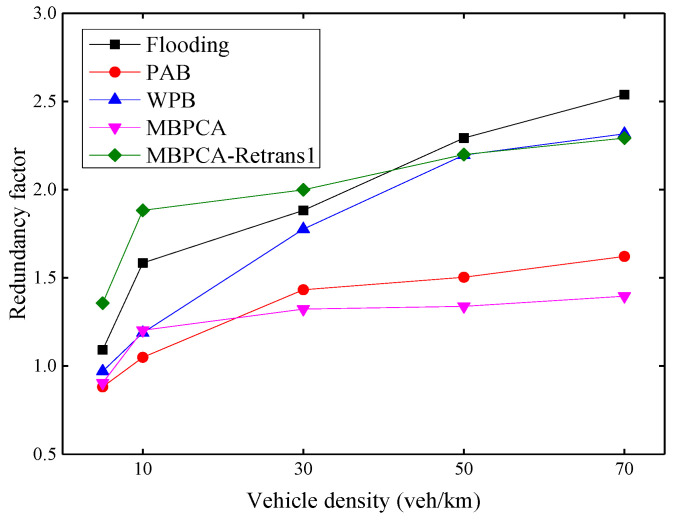
Redundancy factor under different vehicle densities.

Figure 15 shows the redundancy factors of several protocols to evaluate the control effect of broadcast storms. The redundancy factor indicates the degree of message forwarding redundancy in multi-hop broadcasting, and a large redundancy factor may lead to a higher transmission collision probability. In general, the redundancy factor increases with increasing vehicle density. This is because more vehicles mean more forwarding nodes, which will increase the redundancy factor. Both MBPCA and PAB can maintain a low redundancy factor because a candidate forwarder will cancel its redundant forwarding after receiving the forwarding from the other neighbor nodes. MBPCA with one retransmission has a higher redundancy factor, but it can achieve nearly 100% packet delivery ratio and reliability factor. Therefore, we should only use retransmission for a few message types that require an extremely high delivery ratio and reliability in practical applications. In fact, the flooding broadcast will have a much higher redundancy factor than the simulation result if the forwarding collision of Emergency messages is not considered. Because forwarding collision not only reduces the reliability factor, but also reduces the number of redundant packets received by the node. Compared with flooding, WPB can reduce the redundancy factor, but the calculation of the node forwarding probability does not consider the vehicle density, so its improvement is limited. It should be noted that unlike the retransmission mechanism of our proposed MBPCA protocol, the retransmission mechanism of the WPB protocol only sets the forwarding probability to 1 when the forwarding of the neighbor node is not received; it does not forward the same message twice.

## 7. Conclusions

This paper proposes a new MAC protocol and a new multi-hop broadcast protocol for VANETS communication in highway scenarios. First, based on the research on the MAC protocol in the WAVE standard and reinforcement learning, we propose a Q-learning MAC protocol based on detecting the number of two-hop neighbors to reduce the transmission collision probability. Beacon messages and Q-learning are used to train the near-optimal MAC layer CW value under different numbers of contention nodes. Then, for the multi-hop broadcast of emergency messages, the preferred forwarder and the candidate forwarder jointly participate in the forwarding process to reduce the forwarding delay and improve the forwarding reliability. The preferred forwarder is selected by the sender using the distance, direction, speed, and RSSI of neighboring vehicles. All neighbor vehicles in the direction of the emergency message transmission are regarded as candidate forwarders. The forwarder sets an appropriate forwarding waiting time according to the trained MAC layer CW value and the distance from the neighboring vehicle to reduce the forwarding collision probability. Finally, based on the Veins platform, our proposed QMAC-2ND protocol is compared with the modified-WAVE and QMAC in terms of average packet delivery ratio, average single-hop delay, and fairness index. In addition, our proposed MBPCA and MBPCA with one retransmission are compared with the flooding, PAB, and WPB protocols in terms of packet delivery ratio, average end-to-end delay, reliability factor, and redundancy factor. The simulation results show that our proposed two new protocols perform better than related protocols. In future work, we will consider further optimizing the protocols proposed in this paper to make them applicable to more vehicular communication scenarios.

## Figures and Tables

**Table 1:** The structure of the beacon message.

SenderAddress	ReplyAddress	MsgID
SenderPosition	SenderSpeed	SenderDirection
ForwardNeiNum	BackwordNeiNum	Priority

**Table 2:** The structure of the emergency message.

SenderAddress	ForwarderAddress	MsgID
OriginalPosition	TransmitDistance	TransmitDirection
Priority	Forwardinghop	Emergencycontent

**Table 3:** The structure of the neighbor table.

ID	Position	Speed	ForwardNeiNum	AckFactor
RSSI (received signal strength indication)	Direction	LastTime	BackwordNeiNum	ForwardFactor

**Table 4:** Simulation parameters.

Class	Parameters	Value
General	simulation time	300 s
	message size	512 Byte
	carrier frequency	5.89 GHz
	channel model	Nakagami
	bitrate	9 Mbps
	transmission power	20 mw
	RXThresh	−89 dBm
	reference transmission radius R	300m
	a slot time	13 µs
	discount rate γ	0.8
	*T_Set_*	200s
Beacon message	priority	5
	transmission interval	100 ms
	retransmission limit	0
Emergency message	priority	7
	transmission interval	3 s
	retransmission limit	0 or 1

## Data Availability

No new data were created or analyzed in this study. Data sharing is not applicable to this article.

## References

[1] Naik G., Choudhury B., Park J.-M. (2019). IEEE 802.11bd & 5G NR V2X: Evolution of Radio Access Technologies for V2X Communications. IEEE Access.

[2] IEEE (2019). IEEE Guide for Wireless Access in Vehicular Environments (WAVE) Architecture—Redline.

[3] Shah A.F.M.S., Ilhan H., Tureli U. Modeling and Performance Analysis of the IEEE 802.11P MAC for VANETs. Proceedings of the 2019 42nd International Conference on Telecommunications and Signal Processing (TSP).

[4] Cheng J., Cheng J., Zhou M., Liu F., Gao S., Liu C. (2015). Routing in Internet of Vehicles: A Review. IEEE Trans. Intell. Transp. Syst..

[5] Pei Z., Chen W., Zheng H., Du L. (2020). Optimization of Maximum Routing Hop Count Parameter Based on Vehicle Density for VANET. Mob. Inf. Syst..

[6] Wu Q., Zheng J. Performance modeling of IEEE 802.11 DCF based fair channel access for vehicular-to-roadside communication in a non-saturated state. Proceedings of the 2014 IEEE International Conference on Communications (ICC).

[7] Karabulut M.A., Shah A.F.M.S., Ilhan H. The Performance of the IEEE 802.11 DCF for Different Contention Window in VANETs. Proceedings of the 2018 41st International Conference on Telecommunications and Signal Processing (TSP).

[8] Zheng J., Wu Q. (2015). Performance Modeling and Analysis of the IEEE 802.11p EDCA Mechanism for VANET. IEEE Trans. Veh. Technol..

[9] Kloiber B., Härri J., Strang T., Sand S. Bigger is better Combining contention window adaptation with geo-based backoff generation in DSRC networks. Proceedings of the 2014 International Conference on Connected Vehicles and Expo (ICCVE).

[10] Rawat D.B., Popescu D.C., Yan G., Olariu S. (2011). Enhancing VANET Performance by Joint Adaptation of Transmission Power and Contention Window Size. IEEE Trans. Parallel Distrib. Syst..

[11] Amuru S., Xiao Y., van der Schaar M., Buehrer R.M. To send or not to send-learning MAC contention. Proceedings of the 2015 IEEE Global Communications Conference (GLOBECOM).

[12] Wu C., Ohzahata S., Ji Y., Kato T. A MAC protocol for delay-sensitive VANET applications with self-learning contention scheme. Proceedings of the 2014 IEEE 11th Consumer Communications and Networking Conference (CCNC).

[13] Pressas A., Sheng Z., Ali F.H., Tian D. (2019). A Q-Learning Approach with Collective Contention Estimation for Bandwidth-Efficient and Fair Access Control in IEEE 802.11p Vehicular Networks. IEEE Trans. Veh. Technol..

[14] Campolo C., Vinel A., Molinaro A., Koucheryavy Y. (2010). Modeling Broadcasting in IEEE 802.11p/WAVE Vehicular Networks. IEEE Commun. Lett..

[15] Virdaus I.K., Kang M., Shin S., Lee C.G., Pyun J.-Y. A counting-based broadcast model of emergency message dissemination in VANETs. Proceedings of the 2017 Ninth International Conference on Ubiquitous and Future Networks (ICUFN).

[16] Zhu B., Xia W., Song T., Shen L. (2009). Position Based Broadcast Mechanism for Inter-Vehicle Cooperative Warning. Proceedings of the 2009 First International Conference on Information Science and Engineering.

[17] Sahoo J., Wu E.H.K., Sahu P., Gerla M. (2009). BPAB: Binary Partition Assisted Emergency Broadcast Protocol for Vehicular Ad Hoc Networks. Proceedings of the 2009 18th International Conference on Computer Communications and Networks.

[18] Bi Y., Shan H., Shen X.S., Wang N., Zhao H. (2015). A Multi-Hop Broadcast Protocol for Emergency Message Dissemination in Urban Vehicular Ad Hoc Networks. IEEE Trans. Intell. Transp. Syst..

[19] Yoo H., Kim D. (2014). ROFF: RObust and Fast Forwarding in Vehicular Ad-Hoc Networks. IEEE Trans. Mob. Comput..

[20] Rehman O., Ould-Khaoua M., Bourdoucen H. (2016). An adaptive relay nodes selection scheme for multi-hop broadcast in VANETs. Comput. Commun..

[21] Wu C., Ohzahata S., Ji Y., Kato T. (2014). Joint Fuzzy Relays and Network-Coding-Based Forwarding for Multihop Broadcasting in VANETs. IEEE Trans. Intell. Transp. Syst..

[22] Wisitpongphan N., Tonguz O.K., Parikh J.S., Mudalige P., Bai F., Sadekar V.K. (2007). Broadcast storm mitigation techniques in vehicular ad hoc networks. IEEE Wirel. Commun..

[23] Yang Y.-T., Chou L.-D. (2008). Position-Based Adaptive Broadcast for Inter-Vehicle Communications. Proceedings of the ICC Workshops—2008 IEEE International Conference on Communications Workshops.

[24] Sangwan A., Ramaiyan V., Shorey R. (2007). Reliable Multihop Broadcast Protocols for Inter-Vehicular Communication in a Fading Channel. Proceedings of the 2007 2nd International Conference on Communication Systems Software and Middleware.

[25] Barradi M., Hafid A.S., Aljahdali S. (2012). Highway multihop broadcast protocols for vehicular networks. Proceedings of the 2012 IEEE International Conference on Communications (ICC).

[26] Abbasi H.I., Voicu R.C., Copeland J.A., Chang Y. (2020). Towards Fast and Reliable Multihop Routing in VANETs. IEEE Trans. Mob. Comput..

[27] Zeng X., Yu M., Wang D. (2018). A New Probabilistic Multi-Hop Broadcast Protocol for Vehicular Networks. IEEE Trans. Veh. Technol..

[28] Zhang X.M., Yan L., Chen K.H., Sung D.K. (2019). Fast, Efficient Broadcast Schemes Based on the Prediction of Dynamics in Vehicular Ad Hoc Networks. IEEE Trans. Intell. Transp. Syst..

[29] Lyu F., Zhu H., Zhou H., Qian L., Xu W., Li M., Shen X. (2018). MoMAC: Mobility-Aware and Collision-Avoidance MAC for Safety Applications in VANETs. IEEE Trans. Veh. Technol..

[30] Sommer C., German R., Dressler F. (2011). Bidirectionally Coupled Network and Road Traffic Simulation for Improved IVC Analysis. IEEE Trans. Mob. Comput..

[31] Lopez P.A., Behrisch M., Bieker-Walz L., Erdmann J., Flotterod Y.P., Hilbrich R., Lucken L., Rummel J., Wagner P., Wiebner E. Microscopic Traffic Simulation using SUMO. Proceedings of the 2018 21st International Conference on Intelligent Transportation Systems (ITSC).

[32] Varga A. (1999). Using the OMNeT++ discrete event simulation system in education. IEEE Trans. Educ..

[33] Jain R.K., Chiu D.-M.W., Hawe W.R. (1984). A Quantitative Measure of Fairness and Discrimination.

